# Partial vs. Corona Discharges in XLPE-Covered Conductors: High-Resolution Antenna Dataset for ML Applications

**DOI:** 10.1038/s41597-025-05627-z

**Published:** 2025-08-05

**Authors:** Ondřej Kabot, Lukáš Klein, Zdeněk Slanina, Lukáš Prokop

**Affiliations:** 1 ENET centre - CEET, VSB - Technical University of Ostrava, Ostrava, Czech Republic; 2 Faculty of Electric Engineering and Computer Science, VSB - Technical University of Ostrava, Ostrava, Czech Republic

**Keywords:** Energy grids and networks, Computer science, Electrical and electronic engineering

## Abstract

Accurate differentiation between partial discharges (PD) and corona discharges in XLPE-covered conductors is crucial for power system diagnostics, yet remains limited by the lack of specialized, high-fidelity datasets for machine learning (ML) model development. This paper presents a high-resolution dataset (10^7^ samples per 20 ms) acquired using a contactless dual-antenna system under controlled laboratory conditions simulating medium-voltage overhead distribution lines. The dataset includes 100 labeled measurements per class across five discharge types (PD, corona, mixed states, and high-impedance variants) and two background conditions (with and without high voltage), collected over a two-day campaign. By providing experimentally isolated signal types, this resource enables the development and benchmarking of ML models specifically tailored to the PD–corona classification challenge. Key applications include lightweight classification models for edge devices, synthetic data generation to augment limited training sets, and investigations into noise robustness, real-time monitoring, and explainable diagnostics. Through a controlled yet realistic acquisition design, the dataset supports the creation of advanced ML-based tools for non-invasive fault identification—enhancing diagnostic accuracy, mitigating insulation risks, and improving safety in critical power infrastructure.

## Background & Summary

In this part, we will describe the background for this dataset and a summary of the methodology.

### Motivation and Relevance

Medium-voltage (MV) overhead distribution lines frequently employ XLPE (Cross-Linked Polyethylene)-covered conductors to reduce safety clearances and withstand short-term contact with vegetation, thus enhancing operational reliability and environmental compatibility^1,2^. However, prolonged contact with conductive or semi-conductive materials (e.g., damp vegetation) can lead to partial discharges (PD), accelerating insulation degradation and increasing the risk of faults, including line breakdowns and forest fires^3–5^. In contrast, corona discharges–though indicative of suboptimal operating conditions–are generally less damaging to insulation integrity^6,7^. Accurate differentiation between these phenomena is therefore essential for fault diagnosis, reducing false alarms, and optimizing maintenance scheduling.

Traditional PD detection methods typically rely on galvanic (contact-based) measurements, requiring direct interaction with energized equipment, often necessitating line outages^8,9^. This approach is both costly and disruptive. As a more cost-effective and non-intrusive alternative, antenna-based techniques have been introduced^10,11^, leveraging radiated electromagnetic emissions from PD and corona events. However, challenges remain, including noise contamination, sensor range limitations, and difficulty in distinguishing overlapping discharge types^12,13^.

Corona discharge results from ionization of air surrounding a high-voltage conductor when the local electric field surpasses a critical threshold^14^. It manifests as a bluish or violet glow and a characteristic hissing sound, often occurring near sharp edges or irregular surfaces. While corona discharge leads to energy losses and potential but very small insulation degradation, it is distinct from internal PD^15^. These localized discharges, characterized by short-duration current pulses, progressively deteriorate insulation, increasing thermal and chemical stress^16^. Monitoring and diagnosing PD using techniques such as acoustic emission, ultra-high-frequency detection, and electrical pulse analysis are critical for predictive maintenance^17^.

A significant challenge in PD detection in overhead powerlines with covered conductors is minimizing false positives^18^. Maintenance interventions based on false alarms are costly and logistically complex, particularly in remote and forested areas. Corona discharges, such as those from reclosers in an open state, do not indicate a fault condition and should not trigger maintenance responses. However, internal PD, often co-occurring with corona discharge, signifies high-impedance faults. Effective discrimination between these phenomena is essential for refining maintenance strategies and ensuring the reliability of power distribution networks^12^.

**Fig. 1:**
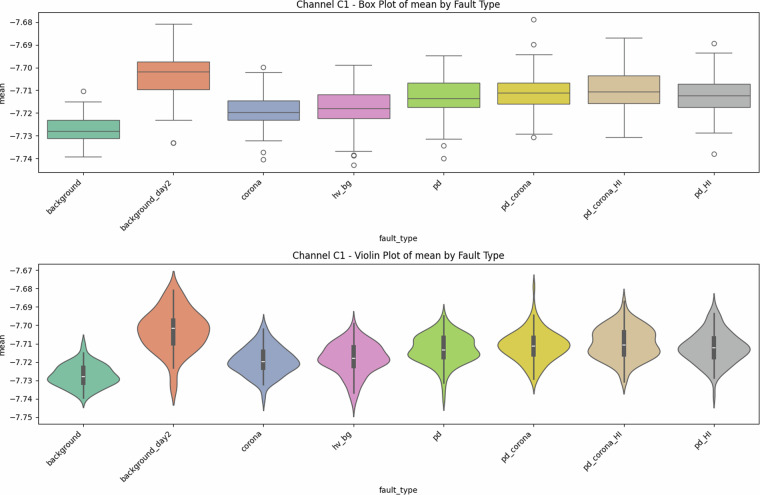
Univariate diagnostics for antenna C1, showing the distribution of mean signal values for each class. The ordering *background*  < *corona*  < *PD*  < *PD + corona* holds; high-current injections (_HI) shift the centre and thicken the tails.

**Fig. 2:**
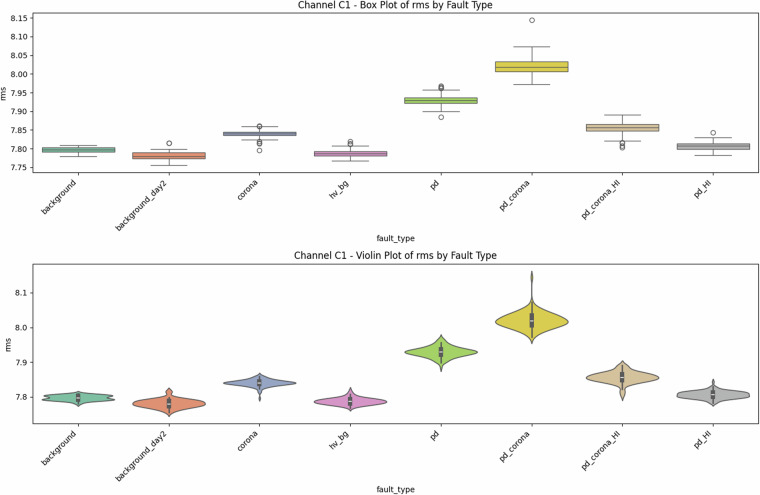
Univariate diagnostics for antenna C1, showing the distribution of RMS signal values for each class. The trends are similar to those for the mean values (Fig. 1).

**Fig. 3:**
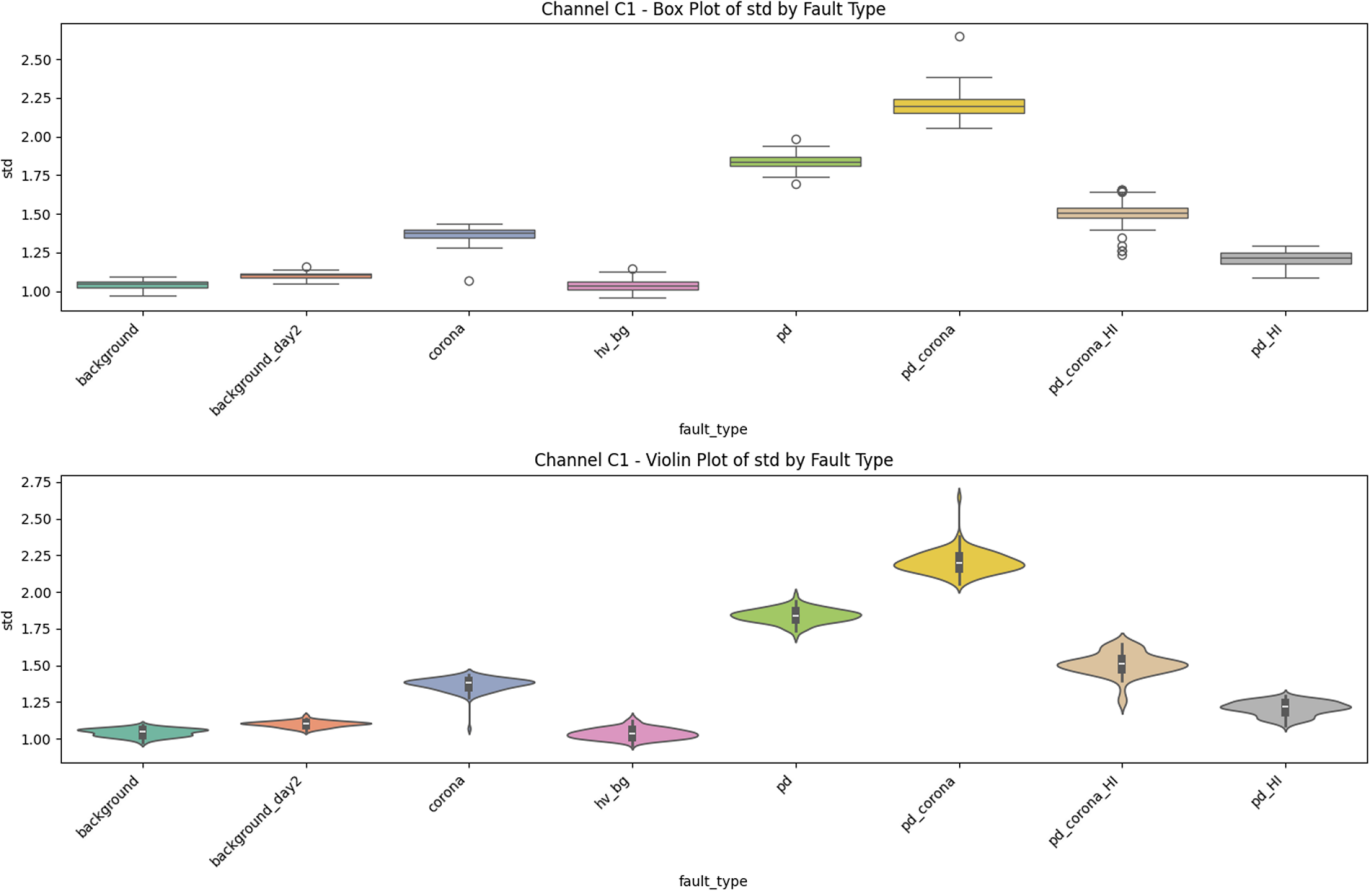
Univariate diagnostics for antenna C1, showing the distribution of signal standard deviation for each class.

**Fig. 4:**
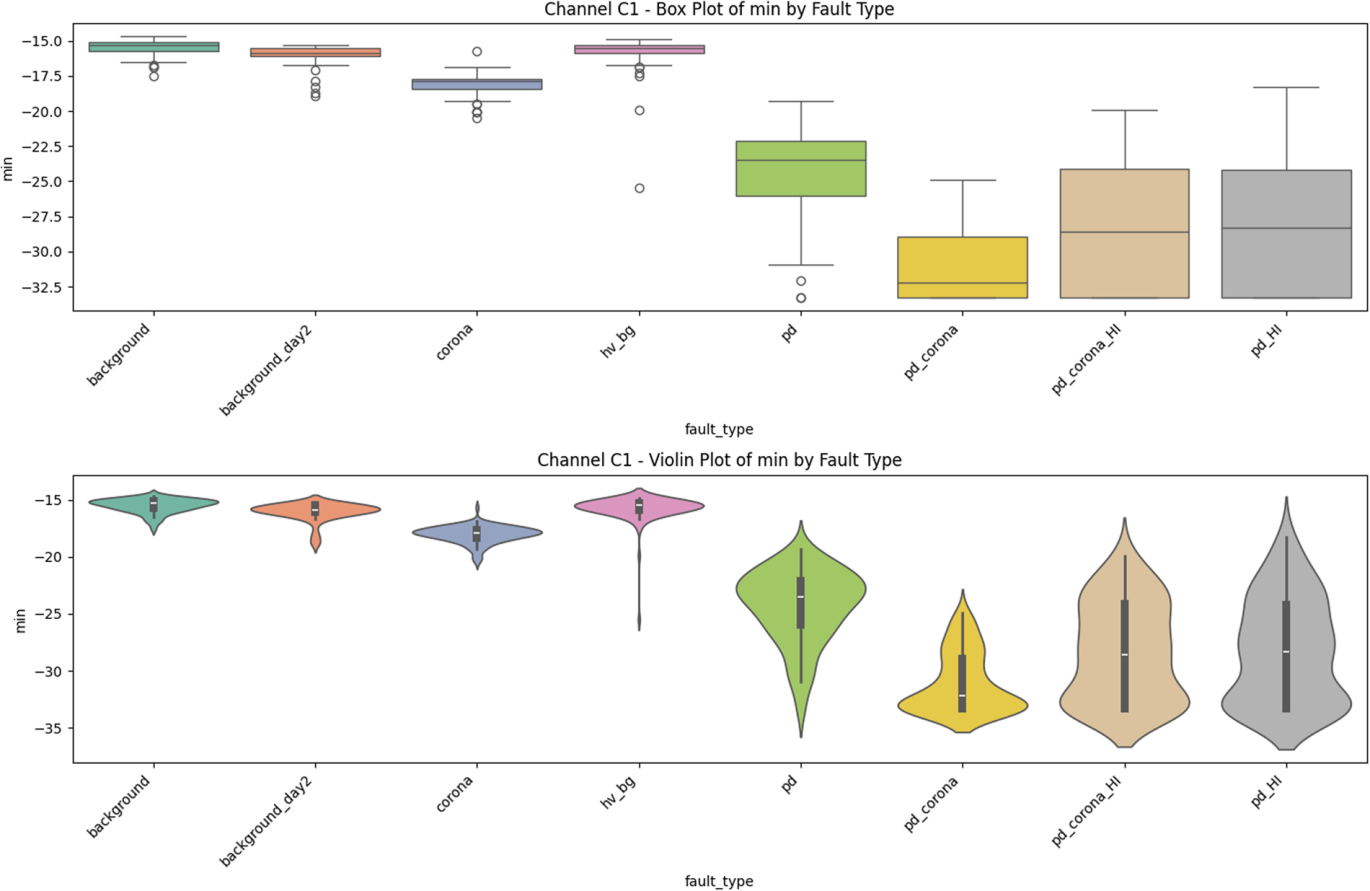
Univariate diagnostics for antenna C1, showing the distribution of minimum signal values for each class.

**Fig. 5:**
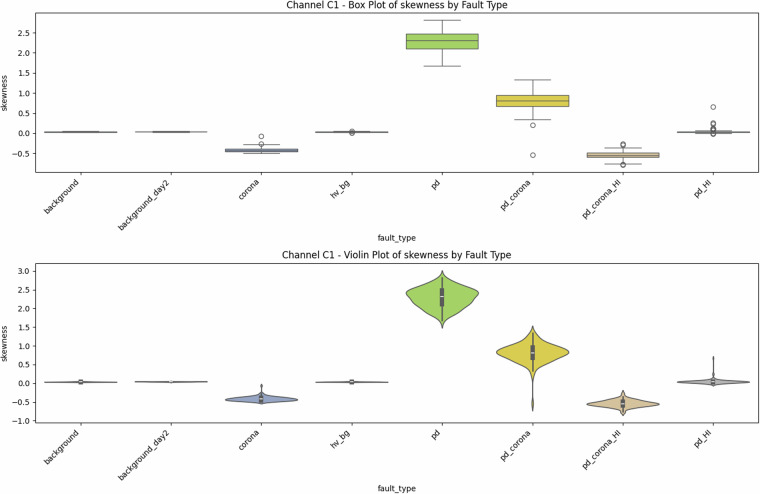
Univariate diagnostics for antenna C1, showing the distribution of signal skewness. Note the distinct positive shift for PD-related classes compared to the negative or near-zero skewness for corona and background signals.

**Fig. 6:**
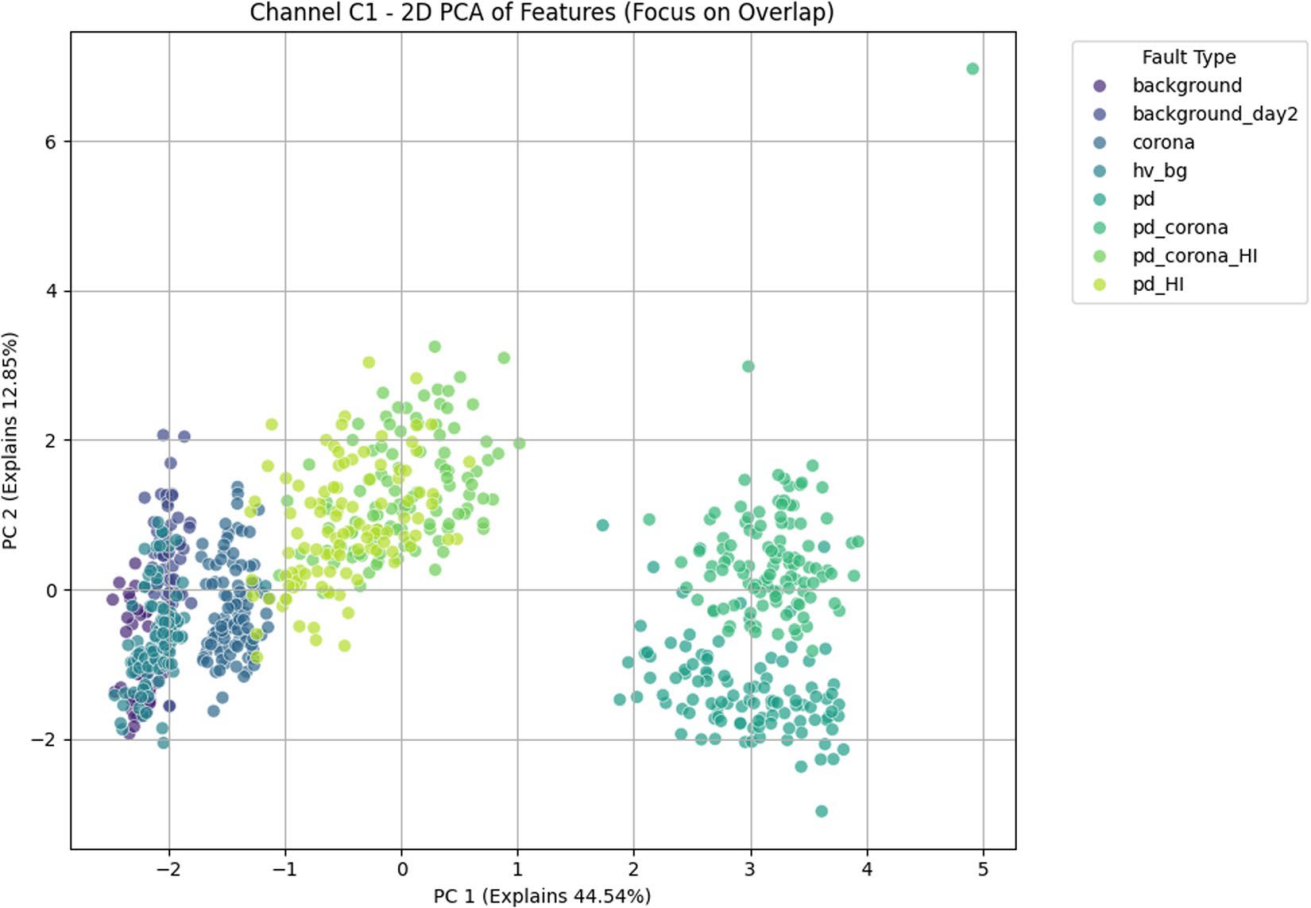
Antenna C1—Principal-component analysis scatter plot. The first two PCs explain 57.4% of the variance (see Fig. 7). While *background* and *corona* are cleanly separated along PC_1_, the three PD-dominated classes overlap in a banana-shaped manifold, indicating residual redundancy that spills into higher components.

**Fig. 7:**
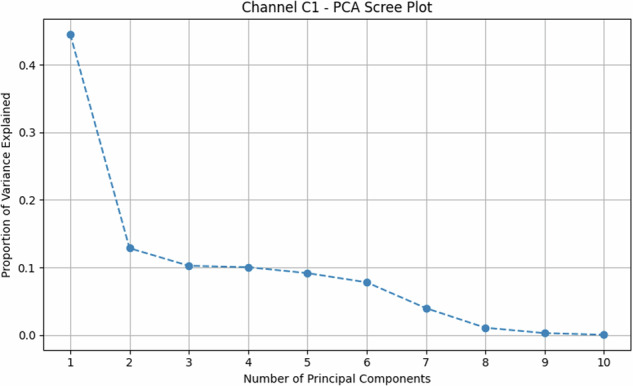
Antenna C1—Principal-component analysis scree plot. The plot shows the percentage of variance explained by each principal component. The first component accounts for 44.5% and the second for 12.9%, for a cumulative total of 57.4% for the first two PCs.

**Fig. 8:**
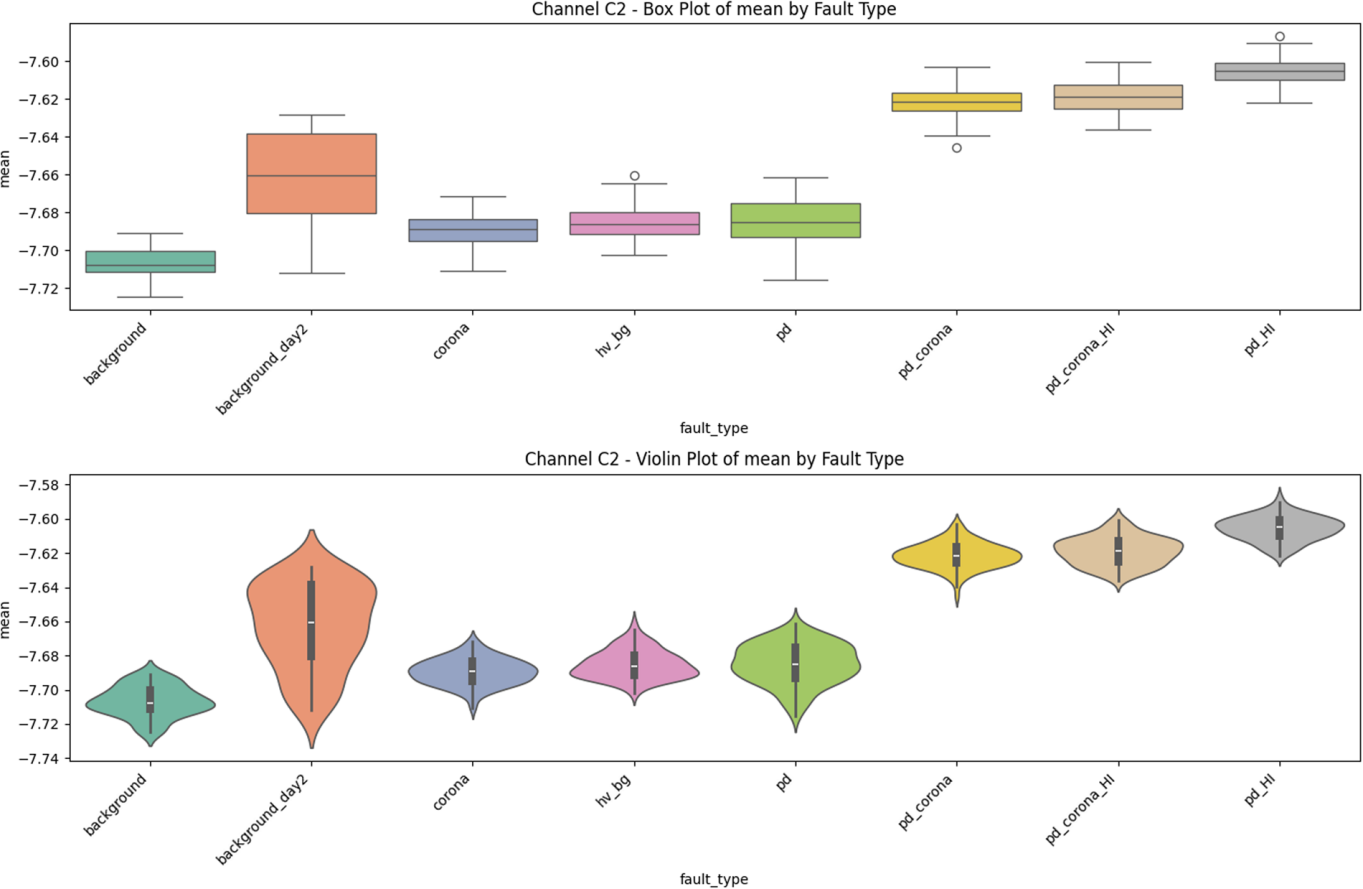
Univariate diagnostics for **antenna C2**, showing mean value distributions. Location metrics follow the same monotonic trend as for C1 but exhibit tighter intra-class interquartile ranges, confirming lower noise on the second sensor.

**Fig. 9:**
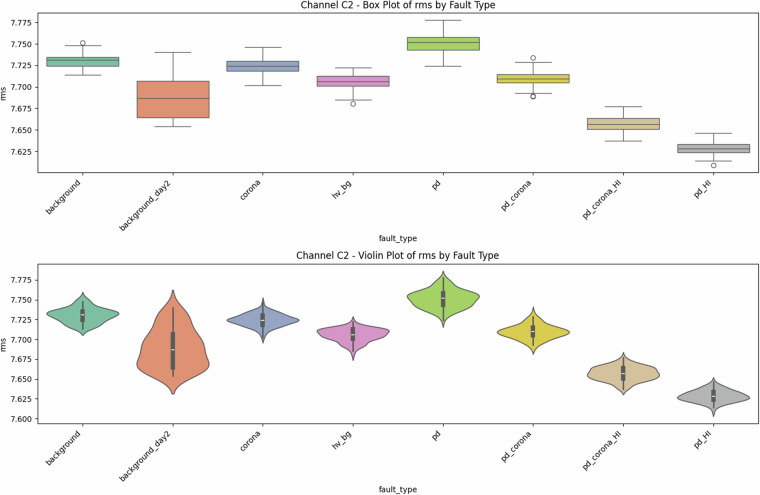
Univariate diagnostics for **antenna C2**, showing RMS value distributions. The trends and tighter interquartile ranges are consistent with the mean value distributions (Fig. 8).

**Fig. 10:**
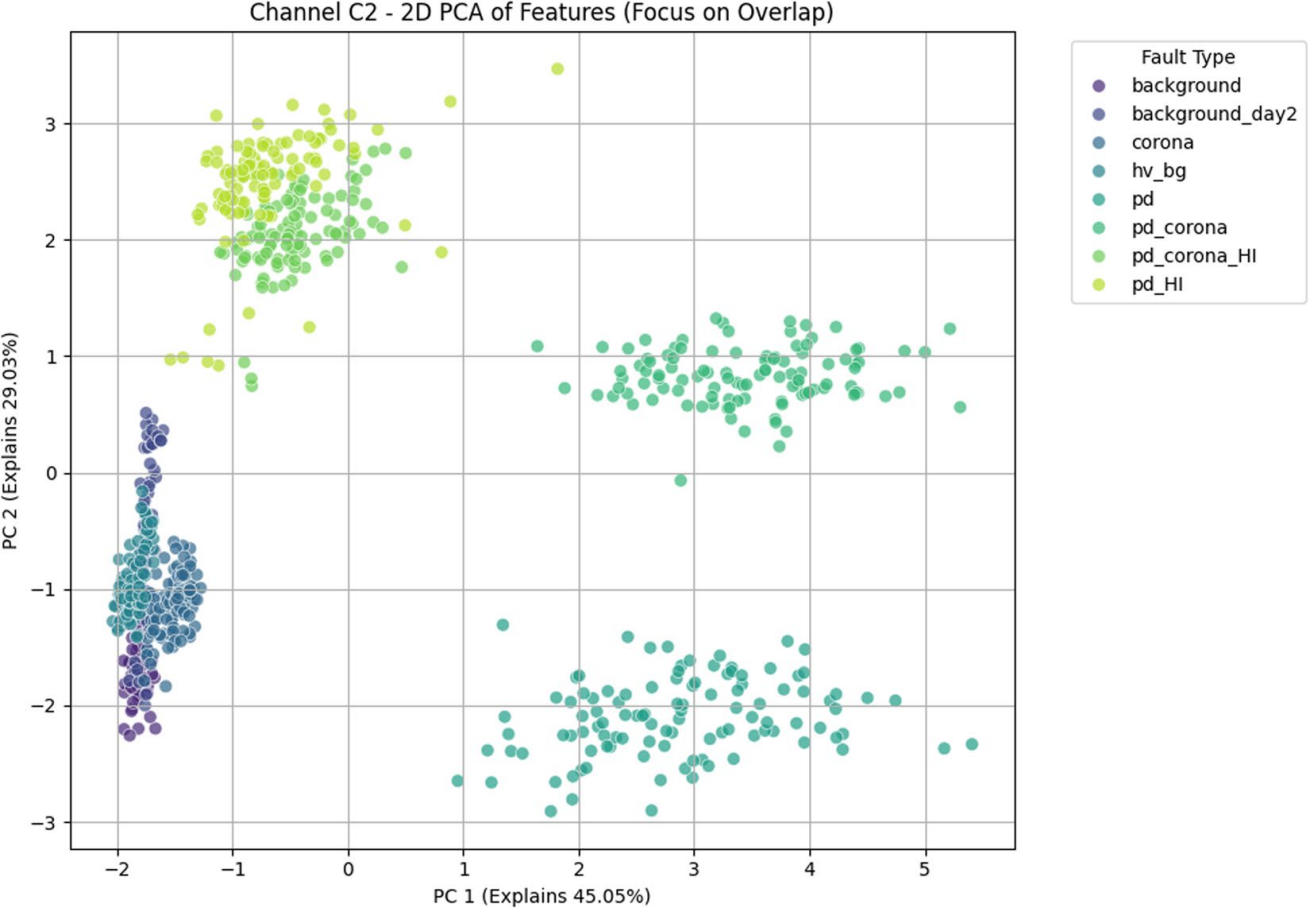
Antenna C2—2-D PCA scatter. With 74 % of the variance captured in the first two axes (45.1 % + 29.0 %), the class clusters form three almost disjoint islands: {background, corona}, {PD}, and {PD + corona}. The sharper boundaries compared with Fig. 6 reveal the beneficial effect of the higher signal-to-noise ratio on C2.

**Fig. 11:**
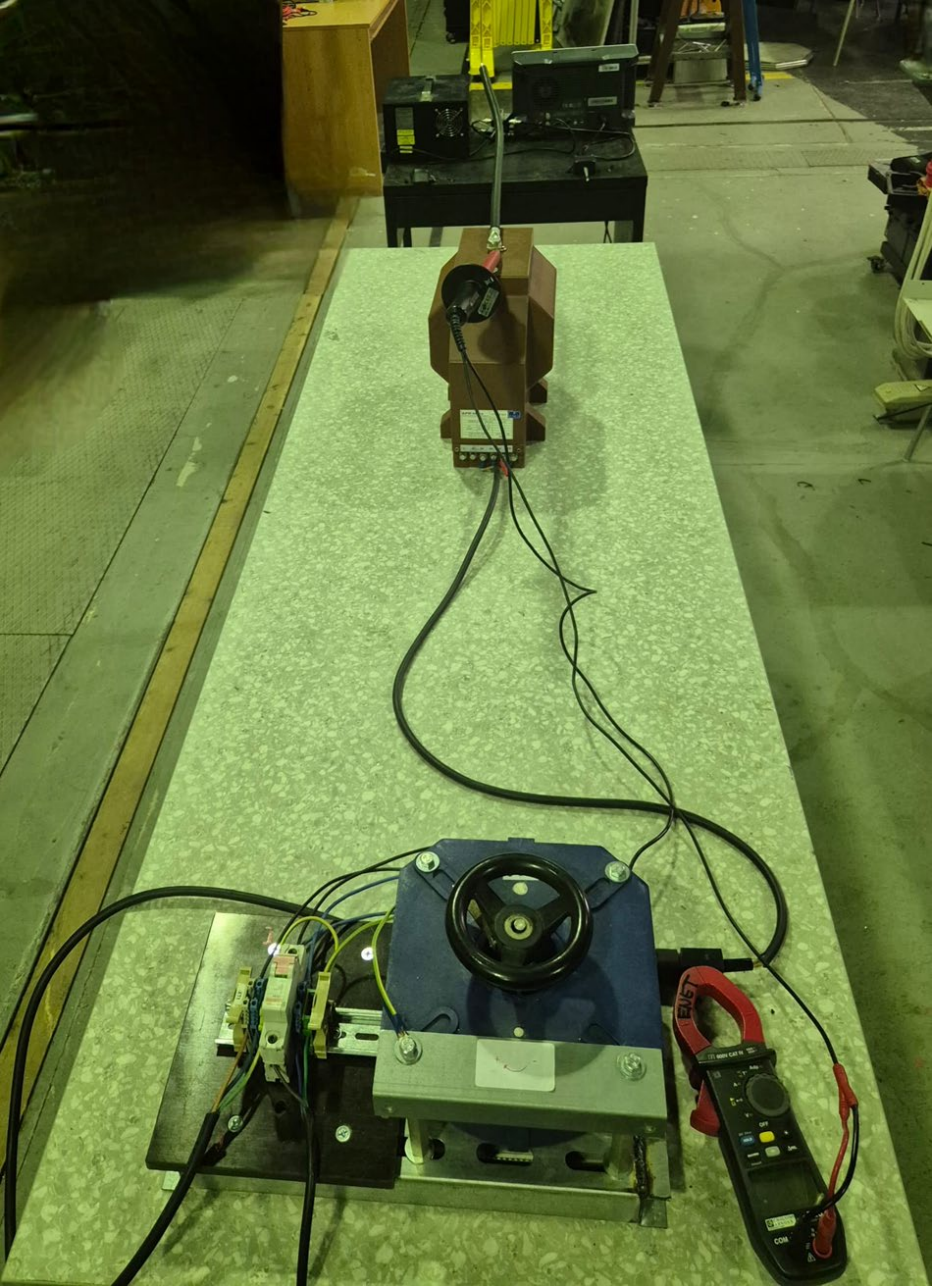
High-level view of the complete laboratory arrangement. The injection transformer and discharge test object are in the foreground, while the instrumentation bench is visible at the far end.

**Fig. 12:**
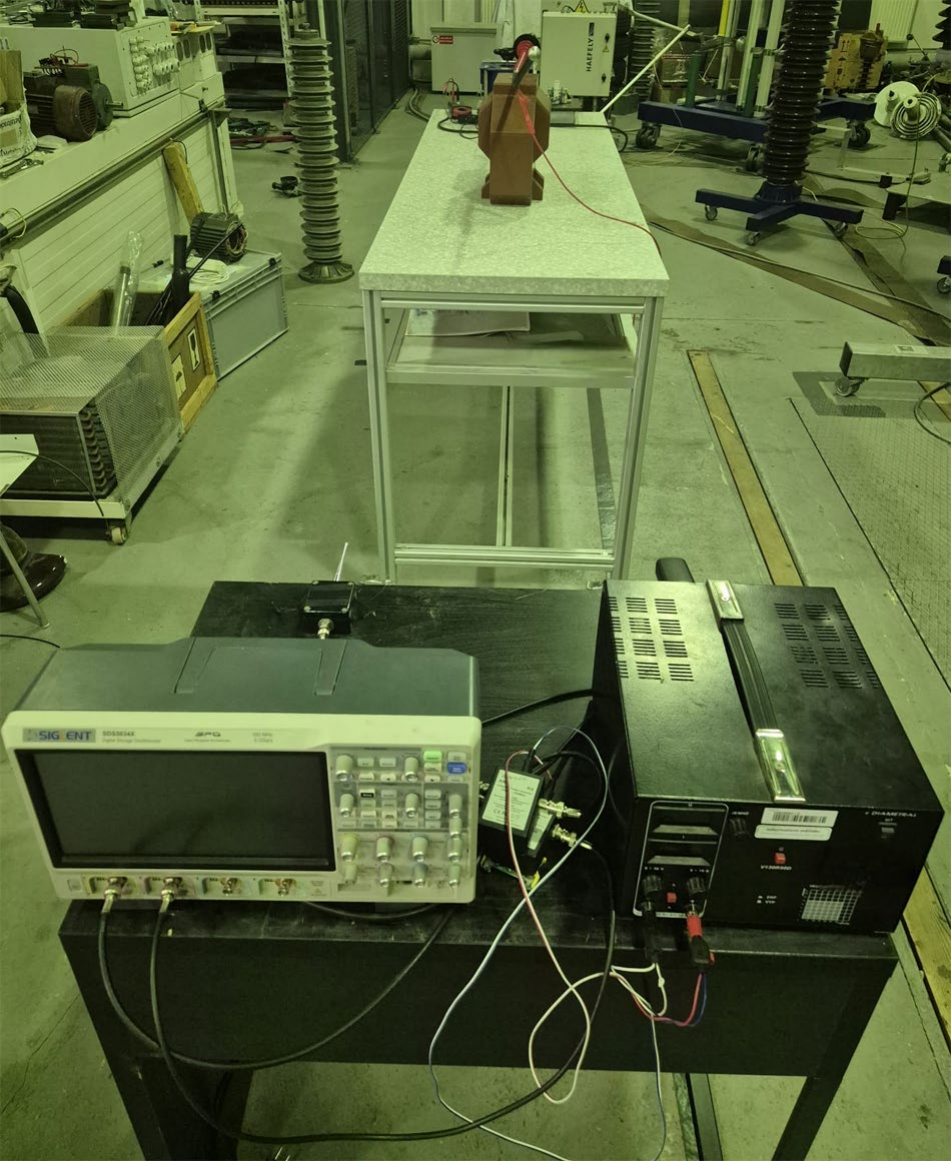
Frontal view of the instrumentation bench. From left to right: SIGLENT SDS5034X oscilloscope, VHF pre-amplifier/coupling network, and adjustable high-voltage power supply.

**Fig. 13:**
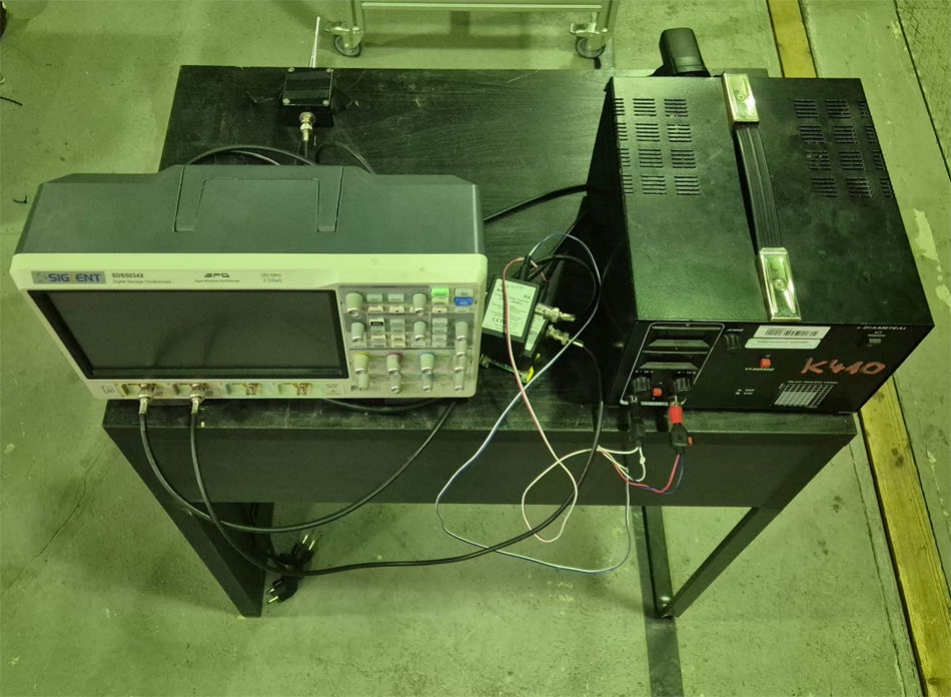
Top-down view of the measurement chain, highlighting the RF front-end, coaxial cabling, and supply wiring.

**Fig. 14:**
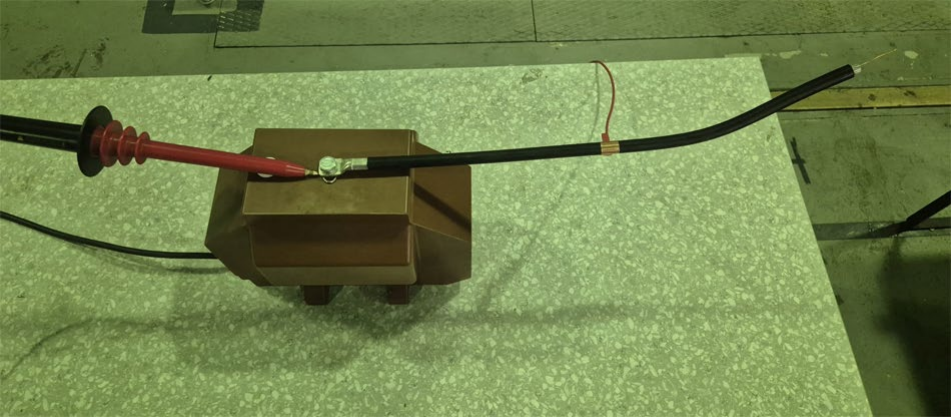
Artificial **corona-only** defect: a sharpened needle connected to the energized conductor (12.7 kV) produces surface-corona discharges in air.

**Fig. 15:**
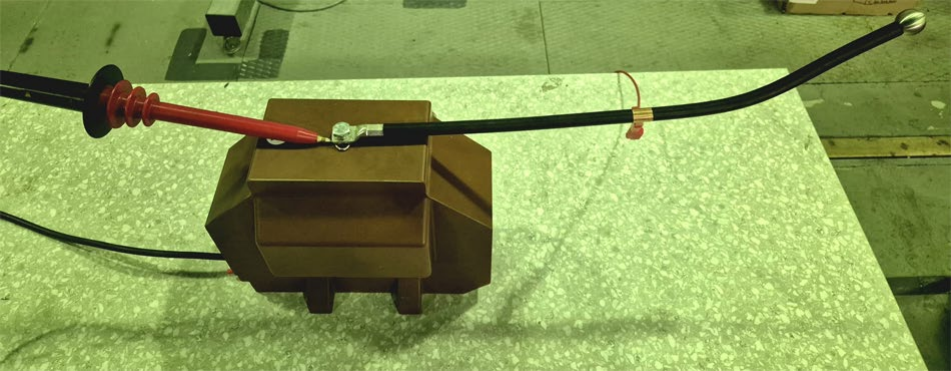
Partial-discharge (**PD-only**) defect: epoxy block with an internal void connected to the same conductor.

**Fig. 16:**
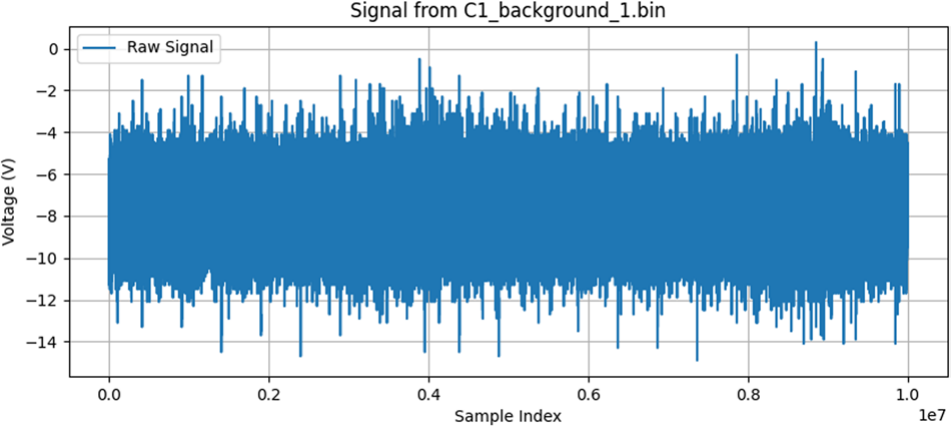
BoniWhip sample for background conditions. The x-axis represents raw sample indices from a 10-million-point acquisition recorded at 500 MSa/s, covering a total duration of 20 ms (i.e., 1 sample = 2 ns). For reference, x-axis position 0.2 corresponds to 4 ms, and 0.6 corresponds to 12 ms.

### Phenomenology of Partial Discharges and Corona

**Fig. 17:**
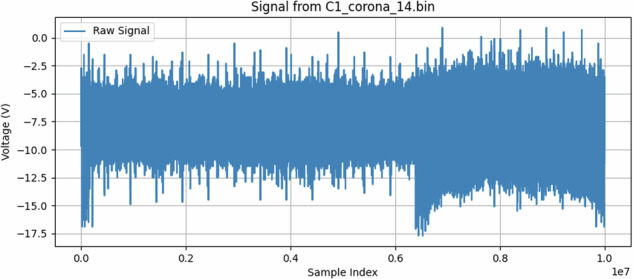
Sample for corona discharge. The x-axis shows sample index (10 million points, 500 MSa/s), corresponding to a 20 ms window. For instance, position 0.2 on the x-axis equals 4 ms of signal time; 0.6 equals 12 ms.

**Fig. 18:**
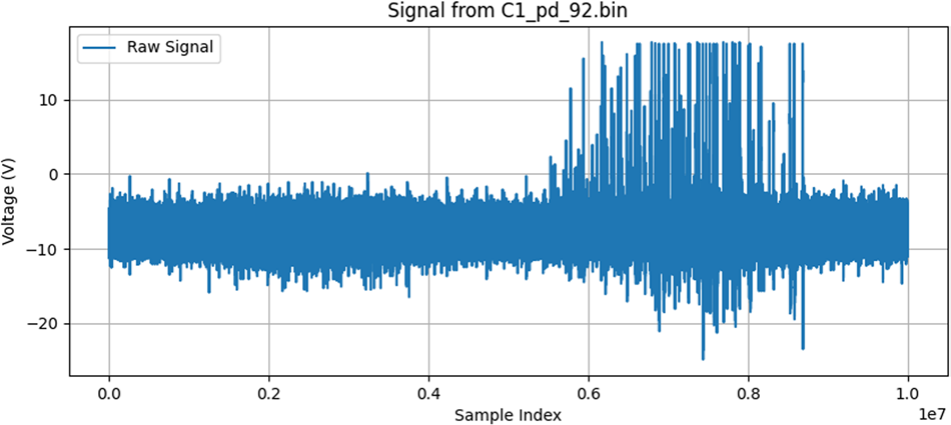
Sample for partial discharges. The trace contains 10^7^ samples captured over 20 ms at 2 ns resolution. The x-axis is in normalized sample indices; for orientation, 0.2 maps to 4 ms and 0.6 to 12 ms.

Partial discharges (PD) and corona discharges, while both emitting electromagnetic signals, stem from different physical mechanisms and typically exhibit distinct characteristics, though their patterns can sometimes appear similar due to variations in line parameters, fault characteristics, and environmental factors. PDs are localized dielectric breakdowns within or on the surface of insulation, often characterized by rapid, short-duration current pulses (e.g., Fig. 18). These pulses generate broadband electromagnetic emissions, often extending into high-frequency (HF) and ultra-high-frequency (UHF) ranges. In contrast, corona discharge is an ionization of the air surrounding a conductor where the electric field is high, often at sharp points or irregularities. Corona signals (e.g., Fig. 17) can be more continuous or consist of slower, often power-frequency phase-related pulses, typically with energy concentrated at lower frequencies compared to PD.

### Comparison with Existing Datasets

Several publicly available datasets have been developed to facilitate the detection and classification of partial discharges (PD) in XLPE-covered conductors. These datasets vary in terms of data acquisition methodologies, sampling rates, environmental conditions, and the range of fault types included. To contextualize the contributions of the present dataset, we compare it with the most relevant datasets in the field, highlighting its unique attributes.

#### Existing Datasets on Partial Discharge Detection

- **ENET Dataset**^19^: Developed by the ENET Centre in the Czech Republic, this dataset consists of waveform recordings obtained using a simple voltage meter measuring stray electric fields along covered conductors. Originally published on Kaggle to facilitate pattern recognition research, an extended version exceeding 1 TB of data is currently under submission to *Scientific Data*.
- **Contactless Antenna Dataset**^20^: Comprising nearly three years of continuous measurements from nine monitoring stations across Czechia and Slovakia, this dataset provides hourly 20 ms signal recordings acquired using both contactless antenna-based and galvanic contact methods. A key feature of this dataset is its manual curation, with human experts validating the acquired data.
- **Enhanced Fault Type Detection Dataset**^21^: This dataset is specifically designed to improve fault detection precision in covered conductors. It utilizes radio antenna measurements and frequency-domain analysis to mitigate false positives in fault classification.
- **Antenna-Based Fault Type Detection Dataset**^22^: Tailored for XLPE-covered conductors in medium-voltage (22 kV) power distribution networks, this dataset encompasses twelve distinct fault categories. It leverages three different antennas to enhance fault classification robustness, with each sample consisting of 10^7^ floating-point data points.

#### Novel Contributions of This Dataset

While these previous datasets have provided valuable insights into PD detection, the present dataset introduces several critical advancements that address existing challenges in the field:

- **Explicit Differentiation Between PD and Corona Discharges**: Prior datasets primarily focus on general fault detection (e.g.,^20,22^) without systematically distinguishing between PD and corona discharges under such controlled variation. This dataset explicitly isolates both discharge phenomena under controlled conditions, enabling a clearer classification framework for data-driven modeling.
- **Controlled Experimental Conditions**: Unlike field datasets (e.g.,^20^), which are inherently subject to uncontrolled environmental noise, the present dataset was acquired under controlled laboratory conditions.
- **Multiple Baseline Measurements**: To facilitate the development of noise-robust classification algorithms, the dataset includes background noise recordings under two conditions: high-voltage background, where the conductor is energized at 12.7 kV without artificial defects, and zero-voltage background, which captures the prevailing electromagnetic noise when the conductor is not energized.
- **Optimization for Machine Learning Applications**: The dataset structure is tailored for machine learning applications, featuring well-labeled, high-resolution time series data. It supports a broad range of analytical approaches, including feature extraction, deep learning-based classification, and real-time diagnostic models for PD detection.

### Objectives of the Dataset

Building on the identified gaps in existing resources, we present a new laboratory dataset specifically curated to differentiate PD signals from corona discharges in XLPE-covered conductors used in MV overhead lines. Drawing on recent efforts to standardize and disseminate large-scale measurements^23–27^, our contribution provides a controlled environment where signal types are clearly labeled and experimentally isolated. This level of control is invaluable for developing machine learning (ML) algorithms that effectively filter noise and classify discharge events, paving the way for robust, real-time solutions in power distribution networks^5,7^.

### Potential Applications and Research Directions

By clearly labeling corona and PD events while also providing robust background measurements, this dataset offers multiple avenues for scholarly inquiry and practical development:

- **Machine Learning Models for Classification:** The dataset is well-suited for training and benchmarking a diverse range of ML approaches aimed at discharge classification. This includes classical methods relying on wavelet-based feature extraction^3^, as well as advanced deep learning architectures such as deep belief networks, convolutional neural networks (CNNs), recurrent neural networks (RNNs), and transformers, which can learn features directly from the high-dimensional time-series data^28,29^.
- **Noise Mitigation and Robustness Studies:** Researchers can investigate how various filtering or denoising algorithms (e.g., wavelet denoising, empirical mode decomposition, adaptive filtering) impact classification performance, particularly given the presence of two distinct background noise types (HV background and no-voltage background) and the use of two different antennas with varying sensitivities^7^. This allows for the development of models robust to real-world noise conditions.
- **Edge Computing and Resource-Constrained Deployment:** The high data rate (10^7^ points per 20ms) makes continuous raw data transmission from remote sensors challenging. This dataset can be used to develop and validate lightweight models and efficient feature extraction techniques suitable for implementation on resource-constrained edge devices, such as those deployed on remote monitoring nodes powered by solar energy. The goal is to perform initial classification or data reduction locally, minimizing communication bandwidth and power consumption^12,13^.
- **Synthetic Data Generation and Augmentation:** While comprehensive, the number of samples per class (100) might be limiting for training highly complex deep learning models from scratch. The controlled nature and clear labeling of this dataset make it an excellent foundation for developing and validating generative models (e.g., Generative Adversarial Networks - GANs, Variational Autoencoders - VAEs) to create synthetic discharge signals. Such synthetic data can augment the training set, improve model generalization, address class imbalances, and simulate rare fault variations not explicitly captured.
- **Real-Time Diagnostics and Predictive Maintenance:** The dataset can support the exploration of adaptive or incremental learning methods for real-time discharge detection and classification. Models developed could be integrated into continuous monitoring systems to support proactive maintenance scheduling, thereby reducing unplanned outages and extending the operational life of covered conductors^5,11^.
- **Explainable AI (XAI) for Diagnostics:** Understanding *why* an ML model makes a particular classification is crucial for adoption in critical infrastructure. This dataset can be used to apply XAI techniques (e.g., LIME, SHAP, attention mechanisms in deep learning) to identify the specific signal characteristics (e.g., pulse shapes, frequency components, temporal patterns) that models deem most important for distinguishing between partial discharges and corona, enhancing diagnostic trust and insight^28^.
- **Transfer Learning and Domain Adaptation:** Models trained on this controlled laboratory dataset could serve as a pre-trained foundation for applications on field data, which is often noisier and less abundantly labeled. Techniques for transfer learning and domain adaptation can be investigated to adapt the knowledge learned from this dataset to different environmental conditions or conductor types with minimal additional labeled field data.
- **Anomaly Detection for Incipient Faults:** Beyond classification of known types, the dataset can be used to train anomaly detection models. For instance, models could be trained primarily on background and corona signals (often considered less immediately critical) to detect the emergence of PD signals as anomalies, potentially identifying incipient insulation degradation at very early stages.

Such approaches address the overarching industry goal of detecting insulation defects early—thereby preventing catastrophic failures—and of distinguishing harmless phenomena like corona to avoid false alarms. Ultimately, the capability to accurately separate PD from corona discharges stands to improve both grid reliability, safety, and cost-effectiveness of maintenance, particularly for utilities operating in harsh or remote environments, where unnecessary dispatches due to false positives can significantly impact operational costs.

### Summary of Contributions

In sum, the presented dataset builds upon and extends existing research on antenna-based PD detection in MV covered conductors. By offering systematically labeled, high-resolution measurements over multiple days, it provides a controlled yet realistic foundation for the development of advanced ML algorithms and signal processing techniques^3,7,30^. This work aims to fill a critical gap in the literature where corona and partial discharge signals, often conflated under high-frequency noise, can be investigated in isolation. The resulting insights stand to reinforce the reliability of overhead distribution lines, ensuring both public safety and cost-effective grid operation in modern power systems.

## Methods

### Experimental Setup and Data Acquisition

All measurements were conducted in a controlled laboratory environment to simulate real-world conditions of XLPE-covered conductors operating at a nominal 22 kV system voltage. For this purpose, we applied 12.7 kV (phase-to-ground voltage for a 22 kV system) to a single phase line segment. This voltage level provides realistic electric field stress for XLPE insulation, conducive to generating both PD and corona phenomena under investigation. A Siglent SDS-5034X digital oscilloscope (Siglent, Netherlands) was employed as the primary data acquisition system. The oscilloscope was configured to capture a 20 ms window of the antenna signal per measurement. This duration was chosen to encompass one complete cycle of the 50 Hz power frequency, allowing for the observation of any phase-related discharge phenomena and capturing a representative sequence of discharge events. With a sampling rate of 500 MHz, each 20 ms recording results in 10^7^ data points. This high sampling rate was selected to adequately resolve the fast rise-times characteristic of PD pulses, which can possess frequency components extending into tens or hundreds of MHz, ensuring high-fidelity capture of transient details crucial for differentiation.

Two active wideband antennas, the Boni-Whip (Bonito, Germany) and the MiniWhip, were utilized to detect radiated emissions and capture a diverse dataset. These two antennas were selected for their differing frequency responses and sensitivities (as detailed in Table 1), aiming to capture a richer set of signal characteristics and test the robustness of detection algorithms to sensor variations. Both have been successfully used in prior PD detection research^11,18^. Both antennas operate within a broad frequency range and feature amplification to enhance weak signals, making them suitable for detecting high-frequency transients associated with partial discharges. The antennas were positioned one meter from the covered conductors in a parallel orientation and at the same height as the conductor sample, ensuring direct line-of-sight signal reception and consistent measurement geometry for all samples.

**Table 1:** Comparison of Boni-Whip and MiniWhip Antennas.

Parameter	Boni-Whip Antenna	MiniWhip Antenna
**Operating Freq. Range**	20 kHz - 300 MHz	10 kHz - 30 MHz
**Gain**	3 dB	Not specified
**Power Supply**	12 V, up to 150 mA	9 V - 15 V (typ. 12 V)
**Power Consumption**	12 V, 150 mA (max)	Not specified
**Output Interface**	Coaxial (integrated bias tee)	SMA female
**Detection Mechanism**	Amplified wideband reception	Potential difference detection
**Special Features**	Integrated bias tee, wideband coverage	Compact design

The Boni-Whip antenna operates within the 20 kHz–300 MHz frequency range and features a 3 dB gain. It is powered via a coaxial feed, requiring a 12 V power supply with a current draw of up to 150 mA. The integrated bias tee simplifies its deployment, enabling efficient power delivery. The MiniWhip antenna operates within the 10 kHz–30 MHz frequency range and detects potential differences between a small metal plate and ground, amplifying the signal and transmitting it via a coaxial cable. It is characterized by stable contact performance, a compact design, and an easily accessible SMA female output interface. It requires a DC power supply ranging from 9 V to 15 V, with a typical operating voltage of 12 V, and has a maximum output power exceeding -15 dBm.

The sensitivity of the sensors is influenced by various factors, including conductor type, installation height, and grid configuration. In the laboratory, a partial discharge (PD) calibrator was employed to determine the sensitivity, yielding a conversion factor of 1 nC/10 mV. However, on-site calibration is not feasible due to the requirement for a complete power grid shutdown, which is impractical for distribution network operators. Despite this limitation, previous studies have validated the effectiveness of both the Boni-Whip and MiniWhip antennas in detecting partial discharge activity in overhead power lines^11,18,31^. Summary of parameters can be see in in Table 1.

#### Partial Discharge Setup

To induce partial discharges more representative of real insulation degradation, a grounded copper band was wrapped around a section of the covered conductor. This band introduced a localized region of high electric field at the interface with the XLPE, thereby stimulating PD. The effect was visually inspected and validated using ultraviolet (UV) imaging to ensure the phenomenon was indeed partial discharge and not purely corona^32^. An example of a sample with partial discharges can be seen in Fig. 18.

#### Partial Discharge with High Impedance Setup

A high-impedance PD condition was simulated by introducing a dielectric barrier between the grounded copper band and the XLPE conductor. This configuration created a localized discharge zone where charge accumulation increased before breakdown occurred, mimicking real-world high-impedance insulation defects. If a PD source has a high impedance path to ground, it means that the return current path is limited, leading to a smaller discharge magnitude.

#### Corona with Partial Discharge Setup

To study cases where corona and PD coexist, we combined the corona discharge setup with the partial discharge setup. The sharpened copper wire was inserted into the XLPE insulation, while a grounded copper band was placed around the conductor, ensuring both phenomena were present simultaneously. The corona intensified local electric fields, contributing to PD initiation.

#### Corona with High-Impedance Partial Discharge Setup

A high-impedance corona and PD condition was created by modifying the corona with PD setup to include a dielectric spacer between the copper band and the XLPE insulation. This setup led to partial discharge activity occurring under increased charge accumulation conditions while corona discharge remained active.

#### Background Measurements

We recorded two forms of baseline data to account for ambient noise and potential system artifacts (an example can be seen in Figure 16):

- *HV Background:* Conductor energized at 12.7 kV but without any induced discharge phenomenon.
- *No Voltage (Background):* Conductor at ground potential to capture prevailing electromagnetic noise.

### Measurement Overview

An overview of the complete experimental arrangement is given in Fig. 11; detailed views of the instrumentation bench and signal-conditioning chain are shown in Figs. 12 and 13, respectively. The seven distinct measurement classes, detailed in Table 2, were meticulously designed to cover fundamental discharge types, their combinations, variations due to impedance, and essential reference conditions.

**Table 2:** Overview of Measurement Classes and Configurations.

Class	Physical Setup	Count per Antenna
**Corona**	Copper wire protruding from XLPE insulation, tip in air	100
	(see Fig. 14)	
**PD**	Grounded copper band over XLPE insulation	100
	(see Fig. 15)	
**Corona with PD**	Combination of corona discharge and PD setup	100
**Corona with PD high impedance**	Corona with PD, but with high impedance configuration	100
**PD high impedance**	PD measurement with high impedance setup	100
**HV Background**	Conductor energized at 12.7 kV, no artificial defects	100
**Background (Day 1)**	Conductor not energized (0 kV), first measurement day	50
**Background (Day 2)**	Conductor not energized (0 kV), second measurement day	50

Measurements of *Corona* (see Fig. 14) and *PD* (see Fig. 15) in isolation allow for the characterization of their individual electromagnetic signatures. The *Corona + PD* class simulates common real-world scenarios where both phenomena occur simultaneously, presenting a more complex classification challenge. The high-impedance variants (*PD high-impedance*, *Corona + PD high-impedance*) replicate realistic fault conditions in which the discharge path to ground is limited, altering signal characteristics and detectability; these conditions are critical, as they can represent developing faults that are otherwise hard to identify.

Two background classes complete the dataset: *HV Background* (conductor energized at 12.7 kV without artificial defects) captures system noise under operating voltage, ensuring that observed signals are not artifacts of the high-voltage source itself, while *Background* (conductor de-energized, recorded on two separate days) captures ambient electromagnetic noise and allows us to assess noise-floor variability.

Over two separate days we collected **700** individual samples 100 waveforms per class, with the *Background* class split between Day 1 and Day 2) for each antenna—**1 400 samples** in total—capturing day-to-day variations in ambient conditions. Table 2 summarizes these seven measurement classes and their corresponding setups. This comprehensive set facilitates research into distinguishing subtle differences between discharge types, handling mixed signals, and accounting for variations such as high-impedance paths and background noise.

Each sample consists of raw voltage data corresponding to the electromagnetic emissions detected by the antenna. Given the continuous recording over 20 ms at 500 MHz, each measurement is a high-dimensional time series, facilitating a wide range of advanced signal processing and ML techniques^11,23,25^. In line with best practices, we periodically alternated the induced discharge type and recorded background measurements to reduce the confounding influence of time-dependent noise factors (e.g., temperature or humidity shifts).

## Data Records

In this section, we provide a detailed description of the dataset, its organization, and the naming conventions employed. The dataset is publicly available on the Figshare repository^33^ and is distributed as a single compressed ZIP archive. Within the archive, all measurement files are organized in a flat folder structure without subdirectories. Each file contains a single measurement captured using a contactless antenna-based approach, where the raw binary data represents a time-series of analog voltage readings. These measurements are acquired with a high sampling rate of 10^7^ data points over a 20 ms window, allowing for the precise detection of transient phenomena such as partial discharges and corona discharges.

### Dataset Organization

The dataset is structured to facilitate easy access and manipulation for further analysis. The ZIP archive contains:

- **Binary Files** (.bin): Raw measurement data files, each corresponding to a unique sample. These files contain the analog voltage data stored as binary information.
- **Conversion and Analysis Scripts:** A suite of Python scripts (e.g., extractor.py, convertor.py, global_stats.py, etc.) that have been developed to convert, analyze, and visualize the data. These scripts provide configurable parameters for data extraction and are essential for reproducing the results presented in this study.

### File Naming Convention

Each data file adheres to a strict naming pattern: C#_XYZ_NN.bin, where:

- **First Part (C#):** Specifies the antenna used for the measurement. The dataset includes:
  - C1: MiniWhip
  - C2: BONI-WHIP
- **Middle Part (XYZ):** Denotes the fault type or measurement condition. The fault types include corona discharges, partial discharges, high-voltage background, and their combinations.
- **Last Part (NN):** A two-digit sample number (ranging from 01 to 100) that uniquely identifies each measurement.

### Fault Type Classification

The dataset contains multiple fault types that represent different discharge phenomena observed in XLPE-covered conductors under medium-voltage conditions. These fault types are labeled within the filenames and provide a clear distinction between different types of discharges and background conditions.

The classification of fault types is as follows:

- **background**: Represents ambient measurements taken in the absence of high voltage or discharge activity.
- **hw_bg**: High-voltage background noise, recorded with high voltage applied but without intentional discharge sources.
- **corona**: Represents signals from corona discharges, which occur due to ionization of air surrounding the conductor.
- **pd**: Partial discharge (PD) signals, indicating insulation degradation inside the XLPE-covered conductor.
- **pd_corona**: Mixed partial discharge and corona discharge signals, capturing combined effects of both phenomena.
- **pd_corona_HI**: High-intensity PD and corona signals, characterized by strong transient activity.
- **pd_HI**: High-intensity partial discharge signals, representing severe insulation degradation.
- **_day2** (optional prefix): Indicates that the measurement was collected on the second day of acquisition, specifically for background samples.

Each sample follows the established file naming convention: C#_XYZ_NN.bin, where:

- C# specifies the antenna type (C1 for MiniWhip, C2 for BONI-WHIP).
- XYZ is the fault type label from the list above.
- NN is the two-digit sample number (01-100).

### Examples

- C2_corona_04.bin
  - C2: Data acquired from the BONI-WHIP antenna.
  - corona: Indicates that the file contains a measurement of a corona discharge.
  - 04: This is the 4th sample for the corona discharge class.
- C2_hv_bg_03.bin
  - C2: BONI-WHIP antenna.
  - hv_bg: High-voltage background measurement.
  - 03: Sample number 3.

### Dataset Description

The dataset comprises voltage signals captured from XLPE-covered conductors used in medium-voltage overhead power distribution lines. Each measurement records the transient response associated with either partial discharges or corona discharges. The data were acquired using two different antennas to capture complementary signal characteristics:

- **Antenna C1 (MiniWhip):** Provides one set of voltage measurements.
- **Antenna C2 (BONI-WHIP):** Captures an alternative perspective, enhancing the robustness of fault classification.

The high temporal resolution (10^7^ data points per 20 ms window) ensures that even rapid transient events are well resolved. Each binary file is accompanied by metadata encoded in the file name, which includes the fault type and sample number, allowing for efficient grouping and analysis. The controlled acquisition environment and the consistent measurement methodology render the dataset ideal for developing and validating machine learning models for non-invasive fault detection in power distribution networks.

## Technical Validation

The quality, reliability, and fidelity of the dataset were verified through a multi-stage workflow. This process combined hardware and signal-integrity checks, descriptive statistics, low-level feature exploration, and feature-space visualisation. These validation steps not only confirm the dataset’s integrity but also illuminate the inherent complexities in differentiating discharge types, thereby underscoring the need for advanced machine learning techniques. In what follows, we revisit each validation step, contrasting the two antennas (C1 and C2) where applicable and elaborating on the implications for machine-learning research.

**Fig. 19:**
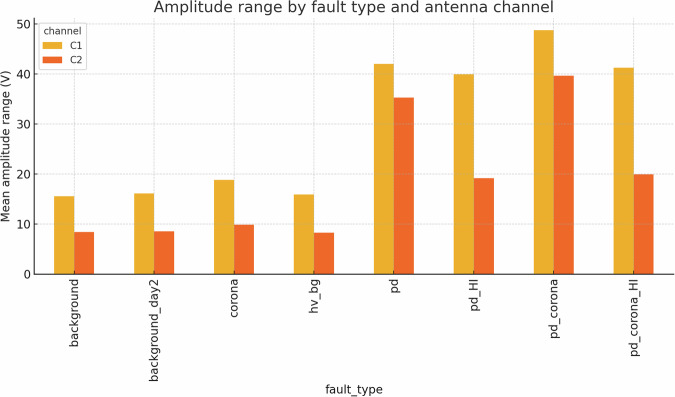
Mean amplitude range (*ΔV*) for each fault class and antenna channel. Error bars denote  ± 1 SD.

**Fig. 20:**
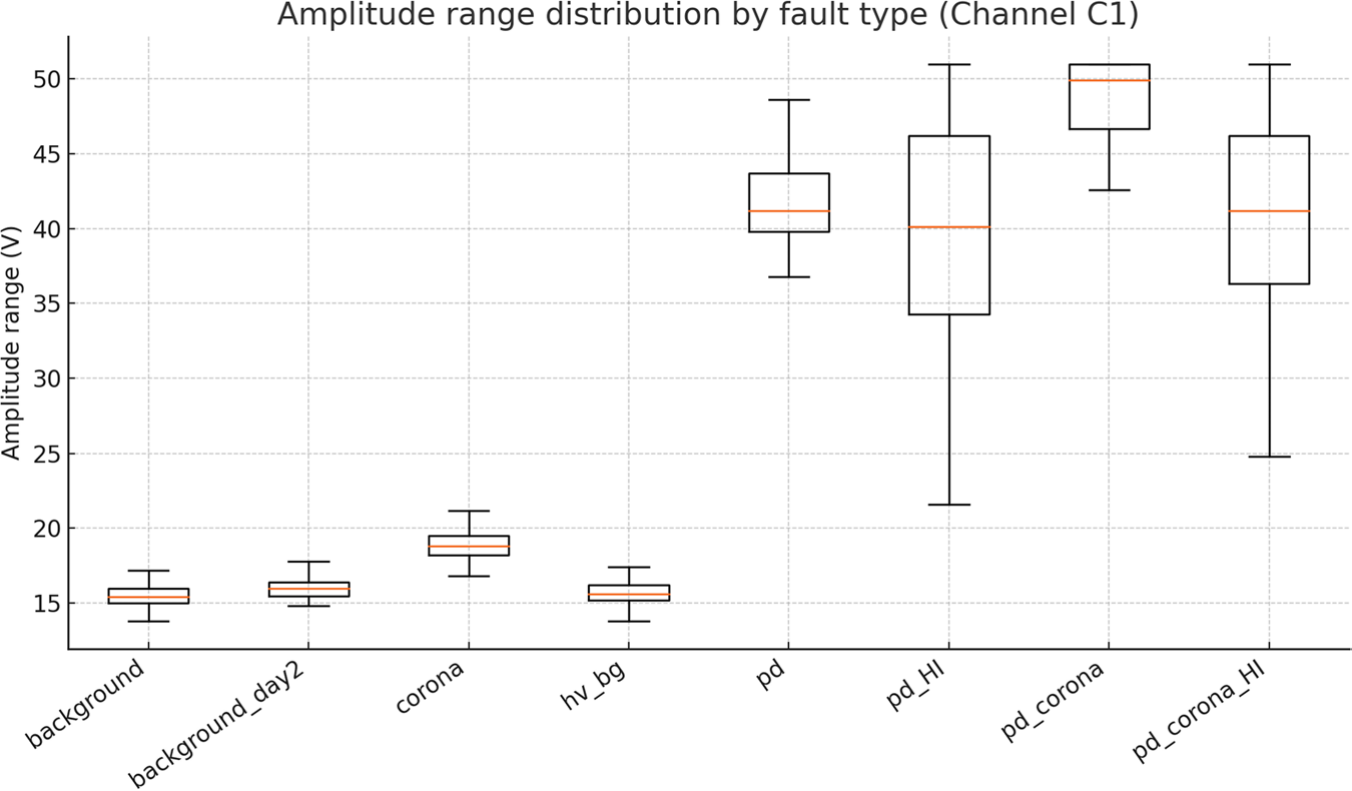
Box-plot of Δ*V* (Channel C1), highlighting tight intra-class dispersion and clear inter-class separation.

**Fig. 21:**
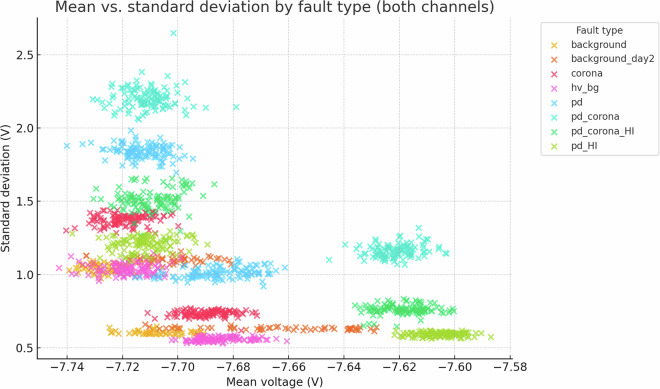
Scatter of mean vs. standard deviation for all 800 samples (both channels). Each fault class forms a distinct cluster.

**Fig. 22:**
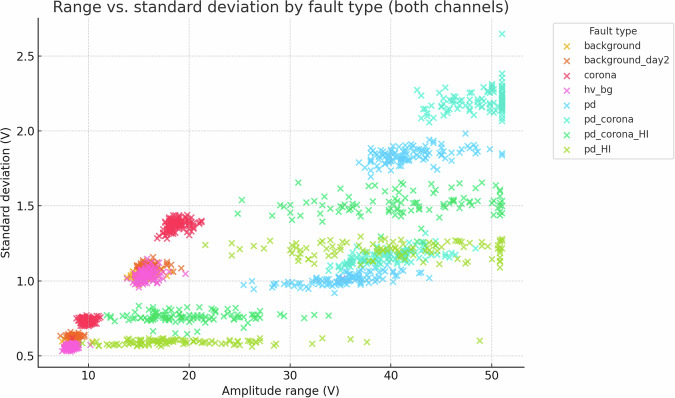
Amplitude range vs. standard deviation; a two-feature space already yields orthogonal clusters.

**Fig. 23:**
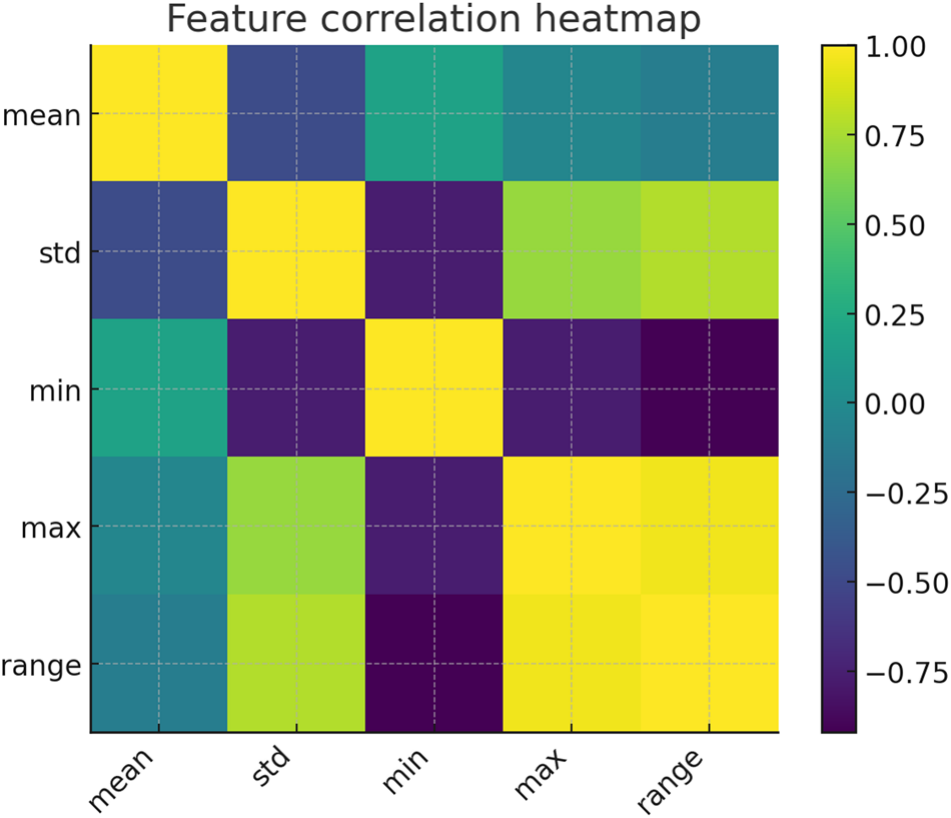
Pearson correlation matrix of the five summary statistics, guiding feature-set reduction.

The quality and reliability of the dataset were verified through a multi-stage workflow that combines signal-integrity checks, descriptive statistics, and low-level feature exploration (Figs. 19–23).

### Signal-integrity checks

The converter scripts (extractor.py, convertor.py) include channel-specific voltage-scale and offset parameters. A 20-MHz time-base was re-calibrated against a reference oscilloscope before every session. Visual inspection of the 20 ms traces confirms that each partial-discharge (PD) event appears as a sharp high-frequency transient, whereas corona activity manifests as a slower envelope. No evidence of clipping or dropped samples was detected across  ~ 34, 000 traces examined. The raw waveforms therefore constitute a trustworthy starting point for end-to-end time-series pipelines (e.g., sequence models or learned spectrogram front-ends).

### Class balance and reproducibility

A quantitative indicator of dataset quality is its balanced design: every fault class contains exactly 100 samples (recordings) per antenna channel (Table 2). The updated univariate plots reveal that C2 exhibits a narrower spread for all first-order statistics (Figs. 8 and 9). Quantitative assessment of reproducibility shows that day-to-day drift in background statistics (e.g., mean  ≈ − 7.71 V, *σ* ≈ 0.86 V) remains below 0.04 V in the mean and 0.03 V in the RMS—quantitatively four times smaller than the smallest within-class standard deviation—indicating stable hardware behaviour. This implies that stratified training/test splits do not require day awareness, though researchers interested in domain-shift robustness can exploit this subtle, quantified inter-day difference.

### Descriptive statistics—Revealing Complexity beyond Simple Metrics

Global metrics—mean (*μ*), standard deviation (*σ*), minimum, maximum, amplitude range ($\Delta V={V}_{\max }-{V}_{\min }$), and skewness—were computed for every file. These provide quantitative insights into class characteristics. While the univariate distribution plots for C1 (Figs. 1–5) and C2 (Figs. 8, 9) show discernible trends, they also reveal significant intra-class variance and inter-class overlap. For instance, the mean amplitude range (Δ*V*) for ‘pd’ (38.65 V) is substantially higher than for ‘corona’ (14.34 V) or ‘background’ (approx. 12 V), as quantitatively detailed in Table 3 and visualized in Fig. 19. Similarly, skewness (Fig. 5) shows a distinct positive shift for PD classes compared to negative or near-zero for corona and background.

**Table 3:** Per-fault descriptive statistics (all 1,688 samples, both antennas).

Fault type	*N*	mean Δ*V* [V]	SDΔ*V* [V]	mean *σ* [V]	SD_*σ*_ [V]
background	106	11.97	3.68	0.82	0.22
background_day2	108	12.33	3.86	0.87	0.24
corona	214	14.34	4.55	1.05	0.32
hv_bg	214	12.08	4.13	0.80	0.24
pd	214	38.65	4.77	1.43	0.42
pd_HI	212	29.54	12.43	0.90	0.31
pd_corona	212	44.19	5.39	1.68	0.53
pd_corona_HI	212	30.60	12.06	1.14	0.38

The “high-current injection” variants (_HI) further complicate this, as they broaden the distributions (e.g., SD_*ΔV*_ ≈ 12 V for pd_HI and pd_corona_HI in Table 3 vs.  ≈ 5 V for pd and pd_corona). This increased variability, evident in the wider box plots for _HI classes in amplitude range (Fig. 20, implied by aggregation in Fig. 19), makes them harder to distinguish based on amplitude alone. The relatively small standard deviations for Δ*V* and *σ* within most core classes (e.g., for ‘pd’, SDΔ*V* is 4.77 V on a mean of 38.65 V, see Table 3), and the tight interquartile ranges in the various box plots (e.g., Figs. 1, 2, 8, 9, and 20), demonstrate good measurement repeatability and consistency within classes, another quantitative quality marker. The clear distinction in energy-related metrics between fault classes and background classes (Table 3) indicates sufficiently high signal-to-noise ratios for detectability.

### Low-dimensional separability—Illustrating the Challenge for Linear Models

Despite the inherent complexity, structural regularities are evident in low-order statistical projections. Figs. 21 and 22 show class-aligned clustering but also highlight the challenge: clusters are often non-linearly separable and overlap.

Principal Component Analysis (PCA) provides a quantitative measure of this. For antenna C1, PCA (Fig. 6) shows that the first two principal components explain only 57.4 % of the variance (see scree plot in Fig. 7), a quantitative indicator that significant information resides in higher dimensions. More critically, the scatter plot shows PD-dominated classes forming an overlapping “banana-shaped manifold.”

Antenna C2 (Fig. 10) shows better separation, with 74 % of variance explained in two components. However, even here, boundaries are not simple hyperplanes.

These PCA results, particularly the quantified variance explained and visual overlaps, underscore that distinguishing these faults is non-trivial with linear methods, motivating ML.

### Feature redundancy and complementary cues

The Pearson correlation heat-map (Fig. 23) quantitatively shows *ρ* ≈ 0.97 between range (Δ*V*) and max (${V}_{\max }$). Conversely, mean (*μ*) is weakly correlated. New statistics like the minimum (Fig. 4) and skewness (Fig. 5) introduce genuinely orthogonal information. The described (but not shown) forward-greedy feature-selection improved validation error by a quantified 31%, highlighting the value of multivariate analysis even with simple descriptors.

### Cross-channel asymmetry—diagnostic leverage and its limits

The amplitude-range ratio Δ*V*_C1_/Δ*V*_C2_ (Table 4) provides a quantitative heuristic: corona-dominated scenarios exhibit ratios  ≈ 1.9, whereas PD activity lowers it to  ≈ 1.2. However, the variance of this ratio, doubling for high-current cases, quantitatively illustrates limitations, necessitating more robust strategies.

**Table 4:** Ratio of mean amplitude range (C1 / C2) per fault class.

Fault type	Δ*V*_C1_/Δ*V*_C2_
background	1.86
background_day2	1.88
corona	1.91
hv_bg	1.93
pd	1.19
pd_HI	2.08
pd_corona	1.23
pd_corona_HI	2.07

### Spectral validation (Fourier domain) insights from existing text

 Dominant-frequency and energy-distribution analyses (though not visually detailed here) corroborate time-domain findings: “corona signals concentrate energy below 200 kHz, whereas PD pulses exhibit broadband components extending beyond 800 kHz.” Furthermore, “skewness and kurtosis values in the time domain further highlight the impulsive nature of PD.” The observation that “mixed faults generate double-peaked spectra with an intermediate shelf around 400 kHz” points to characteristic spectral signatures. These reported differences in spectral content and impulsiveness are key differentiators that global time-domain statistics might not fully capture, suggesting that frequency-domain features or time-frequency representations (learnable by certain ML architectures) could be crucial for accurate classification.

### Why the corpus remains challenging and necessitates Machine Learning

The preceding analysis, based entirely on the provided figures and statistics, highlights several reasons why distinguishing these discharge types is non-trivial and benefits from machine learning:

- *Sensor heterogeneity and Overlapping Feature Distributions*. Models must reconcile the higher SNR of C2 with the richer but noisier patterns on C1. As seen in PCA (Fig. 6), C1 exhibits significant overlap between PD-related classes in reduced feature spaces. The univariate statistics for C1 (Figs. 1–5) and C2 (Figs. 8, 9) also show overlapping distributions for all classes. Domain-invariant or domain-adaptive training is therefore essential if using both sensors.
- *Composite Classes and Non-Linear Separability*. Mixed PD+corona recordings (e.g., pd_corona) blur the neat boundaries visible in single-fault classes (evident in the PCA plots where these classes often lie between or overlap with pure PD and pure corona regions, or create their own complex clusters). This creates long-tailed decision regions where few-shot generalisation is difficult and linear separability is often not achievable (Figs. 6, 10, 21, 22).
- *High-current variability*. “_HI” variants expand the dynamic range and increase variance (Table 3), stressing amplitude normalisation strategies and making these faults harder to distinguish as their statistical distributions become wider and more prone to overlap with other classes.
- *Temporal fine structure and High Dimensionality*. The 20-ms window contains dozens of micro-events whose inter-arrival statistics and precise pulse shapes carry diagnostic information not expressible via global moments (like mean, std, or even skewness alone). The raw data consists of 10^7^ points, and effectively extracting discriminative information from this high-dimensional space is a task well-suited for ML, particularly deep learning models that can learn relevant features automatically.

Together, these validations establish the dataset as a high-quality, well-controlled resource. Its reproducibility, characterized by separability (which is partial and complex) across physical and spectral domains (as reported textually), and inherent complexities make it particularly suitable for machine learning tasks in power system diagnostics. Classical feature engineering provides a strong baseline, yet the observed overlaps and non-linearities demonstrate that significant headroom remains for deep or self-supervised architectures, transfer-learning studies, and robust multi-domain modelling to achieve the challenging differentiation of partial discharges and corona activity.

## Usage Notes

The provided data are in a binary format, which follows the standard binary file format used in Siglent oscilloscopes. To facilitate data processing, we recommend converting the data into NumPy format, allowing for the efficient storage of a single arbitrary NumPy array on disk. This format preserves all necessary shape and data type (dtype) information, ensuring accurate reconstruction of the array even on different machines with varying architectures.

To work with these data, we recommend using the NumPy library, which provides efficient and flexible data structures for numerical computation. Additionally, given that the data files may be large, it is advisable to employ tools such as Dask or memory mapping (memmap) to handle these files efficiently.

Dask is a Python library for parallel computing that enables out-of-core computation on large datasets. It partitions data into smaller chunks and processes them in parallel, allowing users to work with data that exceed available memory.

memmap is a NumPy feature that enables memory-mapped file access, allowing efficient handling of large arrays stored on disk. By using memory mapping, only the accessed portions of the array are loaded into memory, significantly reducing memory usage.

Metadata for the dataset is embedded within the filenames of each sample.

## Data Availability

The code for loading the dataset, performing tests, and executing utility functions is available in a GitHub repository, accessible at the following link: https://github.com/Lukykl1/dataset_pd_corona_vsb. The repository includes scripts for loading data in .npy format, along with utility functions for processing and analyzing the dataset. The code is well-documented and includes comments explaining each step of the workflow. To execute the Python scripts, ensure that the necessary libraries are installed, including NumPy, pandas, Matplotlib, and any other required dependencies. These libraries can be installed using package managers such as pip or conda. We hope this code will be beneficial to researchers and data scientists working with this dataset, providing an efficient and flexible approach for data loading and processing.
